# Approach with initiative or hold on passively? The impact of customer-perceived dependence on customer forgiveness in service failure

**DOI:** 10.3389/fpsyg.2022.914024

**Published:** 2022-08-29

**Authors:** Xin Chen, Shuojia Guo, Jie Xiong, Shuyi Hao

**Affiliations:** ^1^Faculty of Business Administration, School of Business Administration, Southwestern University of Finance and Economics, Chengdu, China; ^2^Department of Marketing, Lucille and Jay Chazanoff School of Business, College of Staten Island, City University of New York, New York, NY, United States; ^3^Department of Strategy, Entrepreneurship and International Business, ESSCA School of Management, Angers, France; ^4^Department of Marketing, Neoma Business School, Reims, France

**Keywords:** service failure, customer-perceived dependence, causal attribution, customer forgiveness, relationship length

## Abstract

Service failure is almost inevitable with the intensifying competition in the service market and expectation of heterogeneous customers. The customer–firm relationship can significantly influence customers’ subsequent attitudes and behaviors to the service provider when they encounter service failure. This study proposes a theoretical model to examine how customer-perceived dependence affects their forgiveness toward a service failure in attribution logic. According to an experiment with 138 and a survey with 428 commercial bank customers, we used a multivariate approach to validate our model. The results show that relationship-valued dependence (RVD) leads to external attribution, which is positively related to customer forgiveness. In contrast, switching-cost dependence (SCD) leads to internal attribution, which is negatively related to customer forgiveness. The relationship length is a relevant contextual factor that acts as a negative moderating factor. Our study contributes to the service recovery literature by elucidating the underlying process of forgiveness with the presence of the customer–firm dependence relationship.

## Introduction

Today, companies strive to provide customers with a better user experience to gain an edge in the increasingly competitive service market (Ramadan et al., 2017; Ho et al., 2020). With the advancement of digital technologies, leading companies are driving innovation and pushing the limits of what can be achieved within their industries. For example, cloud computing, AI/machine learning and blockchain are exciting new technologies that are currently transforming the financial services industry. Nevertheless, the risk of service failure is still inevitable as human error, machine error and system failure are hard to eliminate (Ho et al., 2020; Dao and Theotokis, 2021). Hence, it is crucial to understand how customers would respond in the event of service failures when the service performance falls below their expectations (Ozuem et al., 2021).

Consumer experience is influenced by a series of complicated psychological and brain responses (Balconi et al., 2021). As an inevitable yet influential phenomenon in the service context (Zhu et al., 2021), understanding the mechanism through which consumers cope is crucial for the service provider. When service failure occurs, customers experience disconfirmation (Smith et al., 1999; Ho et al., 2020), which triggers an internalizing coping process to mitigate the stress associated with the negative experience (Sengupta et al., 2015; Tan et al., 2021). The common strategies include avoidance, support-seeking, and forgiveness (Yagil and Luria, 2016; Muhammad and Gul-E-Rana, 2020). Most existing studies focus on avoidance and support-seeking, while customer forgiveness received less attention. Recently Sarofim et al. (2022) investigated how religiosity affects customer forgiveness and found that religiosity corresponded with higher levels of belief-in-fate, leading to lower customer dissatisfaction and higher customer forgiveness. Customer forgiveness is a deliberate and controllable process during which customers reduce their anger and forswear the intention to revenge. While some studies argued that the customer–firm or customer–brand relationship is a crucial antecedent of forgiveness (e.g., Balaji et al., 2016; Tan et al., 2021), there is no consensus on how such a relationship would affect forgiveness.

Additionally, as service competition intensifies, service providers build up switching barriers such as relational benefits or switching costs to maintain customer relationships characterized by customers’ psychological or behavioral dependence (Al-hawari, 2014). The importance of dependence in the B2C customer–firm context has rarely been studied, especially in its linkage with customer forgiveness (Yagil and Luria, 2016; Najafi-Tavani et al., 2020). To address such research gaps, we examine how customer-perceived dependence affects forgiveness through different causal attributions in the face of service failure, moderated by the longevity of their relationship with the firm.

Our findings suggest that relationship-value dependence (RVD) is more likely to trigger external attribution that positively leads to forgiveness, whereas switching-cost dependence (SCD) triggers internal attribution that negatively impacts forgiveness. Overall, the effect of dependence on forgiveness is negatively moderated by the length of the relationship and through the mediating mechanism of causal attributions. Specifically, customers with a value-based dependence will act less willing to forgive when facing a long-term relationship, while customers in a cost-restricted dependence relationship will be more tolerant to give forgiveness after a service failure.

Our study contributes to the service recovery and relationship management literature. First, we elucidate the underlying process of forgiveness with the presence of the customer–firm dependence relationship. Second, our study promotes the theoretical understanding of dependence by discriminating the relationship-value dependence (RVD) along with switching-cost dependence (SCD). Finally, we contribute to the service literature by bridging the research gap between post-transgression reactions and causal attribution processes grounded in relationship contextual factors.

## Theoretical underpinning and literature review

### Customer forgiveness in service failure

Customer forgiveness is often defined as a process to reduce anger, the willingness to foreswear revenge and the enhancement of compassion and generosity to the involved service provider after a service failure. Prior studies have supported its multi-dimensional structure with cognitive, affective and behavioral components (Tsarenko and Tojib, 2011; Muhammad and Gul-E-Rana, 2020; Kravchuk, 2021) and mainly focus on trust, satisfaction, word of mouth, and behavioral intention, meanwhile overlooking forgiveness as a positive response to the service provider (Honora et al., 2022). A large body of research on forgiveness has examined its configuration and determinants. Finkel et al. (2002) suggest the mental state factors of the forgiving party and the ongoing relationship features in which forgiveness occurs are the two vital elements determining forgiveness. Tendencies of revenge decrease when a greater value is placed on the relationships (Zechmeister et al., 2004). The forgiveness process will be influenced by situational or contextual factors, such as contingent factors considered in the causal attribution, characteristics of the relationship between customer and service provider (Tsarenko and Tojib, 2012; Riaz et al., 2016; Muhammad and Gul-E-Rana, 2020; Kravchuk, 2021; Yang and Hu, 2021). For example, customers tend to forgive a service failure due to unforeseen, irresistible factors beyond the service provider’s control (Nikbin et al., 2016). They are also likely to forgive a service failure with timely and appropriate remedial measures (Siu et al., 2013). In addition, the service provider’s integrity, competence, benevolence and awe can lead to customer forgiveness and repair the trust (Xie and Peng, 2009; Yang and Hu, 2021). However, empirical findings on how relationship characteristics moderate forgiveness in service failure are inconsistent. Some studies suggest that the friendliness of relationships and psychological resilience of customer has a positive association with forgiveness (Yagil and Luria, 2016; Kravchuk, 2021), while others find the results to be insignificant or even opposite (Wan et al., 2011; Wang and Wu, 2012; Muhammad and Gul-E-Rana, 2020). The conflicting findings reinforce the importance of an integrative conceptual framework in identifying the boundary conditions that govern customers’ forgiveness of service failure.

### Attribution theory

Attribution theory is a widely applied social cognition theory to explain customers’ thinking and acting processes in marketing filed (Fu et al., 2021). When customers encounter a service failure, they will look for the underlying cause and experience specific emotions, attitudes, or behavior to the service provider. According to the attribution theory, locus, stability, and controllability are the critical constructs of causal attribution (Hess, 2008). In a service failure context, locus refers to whether the cause of failure results from the service provider or customers themselves; stability indicates whether the failure is comparatively enduring or relatively temporary; and controllability is associated with whether the cause of failure is within the firm’s control (Folkes, 1984; Weiner, 2000). Consumers’ causal attribution direction impacts their overall evaluation of the service (Van Raaij and Pruyn, 1998) as well as their reasoning styles (Yoon, 2013). They will consider the attribution of the locus as a primary concern and then attribute stability and controllability accordingly. Thus, in our study, we focus on the attribution of locus and investigate two attribution conditions-internal and external attributions. External attribution attributes the service failure to be at fault of the service provider, while internal attribution attributes the service failure to be out of control of the service provider. Different from prior studies that examine external and internal locus between the service provider and customer, we form a dichotomy from the relationship management perspective focusing on the role of the offending party, considering only if the cause is located on the side of the service provider or not.

Extant literature has shown that different attributions will influence customers’ emotions, evaluations, attitudes and behaviors toward the service firm (Nikbin et al., 2016; Fu et al., 2021). For instance, customers are more likely to show negative responses, such as anger, dissatisfaction, avoidance, revenge, or switching behaviors, when they perceive the failure to be enduring or the firm with the ability to control but failed to do so (Fu et al., 2021). In addition, they are more likely to forgive when perceiving the cause of service failure as something out of the service provider’s hand (Nikbin et al., 2016; Su et al., 2020). Literature has also found the causal attribution intentions associated with contextual elements such as failure types, failure severity, previous service experience, customer knowledge, customer expectation, relationship quality, relationship types and so on (Chen and Huang, 2015; Yagil and Luria, 2016; Su et al., 2020; Fu et al., 2021).

### Grand theory of dependence

Dependence is key to establishing a long-term relationship (Waheed and Gaur, 2012) and for the relationship to yield desirable outcomes (Sung and Choi, 2010). It emphasizes a customer’s need to maintain a relationship for achieving his or her desired goals (Drigotas and Rusbult, 1992; Scheer et al., 2015). In relationship marketing literature, dependence, trust and commitment are the determinant variables for establishing a long-term relationship (Waheed and Gaur, 2012). In the service context, customer trust reflects the belief that customers consider the service provider will not break its promise. Commitment is described as the enduring desire to maintain a valued relationship in order to obtain greater benefits in the future (Waheed and Gaur, 2012). While both trust and commitment reflect a customer’s active intent to build a relationship and the confidence to receive positive outcomes from that relationship, dependence on the other hand emphasizes a customer’s need to retain a relationship with a specific supplier to gain access to particular resources (Najafi-Tavani et al., 2020). Dependence typically arises from the irreplaceable resource customer cannot abandon or the difficulty in accessing alternate outcomes (Balaji et al., 2016). When customers are relatively dependent on their service providers, they are more likely to use outcome-based criteria to evaluate services, in which rational context trust and commitment may have less impact on their subsequent behaviors (Najafi-Tavani et al., 2020).

Relationship literature asserts that customers and service providers strengthen their existing connections through relationship investment in each service encounters (Balaji et al., 2016). Relationship investment not only offers customers additional benefits and value, but also produces barriers or costs for customers’ switching behavior (Al-hawari, 2014; Pick and Eisend, 2014). Based on motivation theory, relationship benefit and switching cost are the two crucial elements for a person-firm relationship, and furthermore drive the positive or negative motivation of customers to establish and maintain their business relationship with the service provider (Scheer et al., 2010). Thus, the nature of dependence customers perceived can be based on positive motivation due to the inherent benefits or based on negative motivation such as switching costs (Scheer et al., 2010; Najafi-Tavani et al., 2020). Following Scheer et al. (2010), our research adopts their decomposition of the overall customer-perceived dependence into two components, namely the relationship-value dependence (RVD) and switching-cost dependence (SCD). The former concerns a customer’s need to maintain a relationship with a service provider due to the unique value or irreplaceable benefits associated with its core offering or operations capability. The latter refers to the need to maintain a relationship due to the dormant costs incurred upon relationship termination. These two dependence components give expressions to the distinguishable sides of a customer’s motivational investment, and they can exist at the same time. Examples of RVD operationalizations include individual customized services, loyalty rewards, preferential treatments like receiving price discounts, faster or additional service, and emotional attachment with the service provider; while examples of SCD operationalizations include pre-investment loss, or anticipated costs of searching, selecting, evaluating, soliciting or transiting to a new alternative (Scheer et al., 2010, 2015). In sum, RVD incarnates the positive motivation to continue the present relationship to obtain relational benefits, whereas SCD is associated with the negative motivation to maintain a relationship due to obstructive barriers that would incur at relationship termination.

In addition, most dependence studies focus on the organizational-level factors from a B2B perspective, including the degree and symmetry of dependence and their impact on subsequent factors (e.g., performance). However, dependence is also common and important in the B2C context. The formation mechanism of the dependence relationship will significantly affect customers’ attitudes and behaviors toward the service provider, but few researchers pay attention to it. Our paper is among the first attempts that studies the distinctiveness of dependence from B2C customers’ perspective, as well as explores its impacts on customer forgiveness from the attribution perspective when they encounter a service failure.

## Hypotheses development

### Causal attribution and customer forgiveness

Extensive studies show that different attributions of service failure will influence customers’ emotions, evaluations, attitudes, and behaviors toward the firms (Nikbin et al., 2016; Su et al., 2020; Fu et al., 2021). Customers are more likely to forgive when they perceive the cause of a service failure to be something out of a service provider’s hands (Su et al., 2020). When they feel the cause is ambiguous or beyond the service provider’s control, the negative emotions or responses will also be mitigated (Choi and Mattila, 2008; Fu et al., 2021). Conversely, when customers make the fault attribution toward firm’s factors like employees, service ability, service system, etc., they are more likely to show negative responses, such as anger, dissatisfaction, avoidance, revenge, or switching behaviors (Nikbin et al., 2016). Moreover, customers react more negatively when they believe the service firm can prevent the failure but fails to do so (Yagil and Luria, 2016). Therefore, we propose:

*H1*: External attribution is positively related to customer forgiveness.

*H2*: Internal attribution is negatively related to customer forgiveness.

### Customer-perceived dependence and causal attribution

RVD and SCD correspond with two distinct types of customer motivational investment. The two types of dependence can be simultaneously high and low, or one dominates the other. RVD incarnates positive motivation to continue the present relationship for obtaining relational benefits, whereas SCD is associated with negative motivation to maintain a relationship due to obstructive barriers that would incur at relationship termination (Scheer et al., 2015).

Causal attribution is an important antecedent for customer forgiveness, expectation, and relationship quality (e.g., Chen and Huang, 2015; Su et al., 2020). In the customer–firm relationship context, customers with RVD can be regarded as owning high expectations due to the irreplaceable unique benefits received in past business encounters (Scheer et al., 2010, 2015). The higher the expectations, the more inconsistent the service failure seems to be with their previously held beliefs. Consequently, customers will tend to make causal attributions that align with their prior beliefs to resolve the dissonance caused by the service failure experience (Fu et al., 2021). Therefore, we posit that RVD and SCD would lead to differential causal attributions to affect customer forgiveness further:

*H3*: Relationship-value dependence is positively related to external attribution.

In contrast, customers with SCD may not necessarily hold high expectations but aim to avoid implicit costs when they remain in the relationship with the service provider (Scheer et al., 2010, 2015). Power imbalance exists in an exchange relation when there is the potential for exploitation. It determines the magnitude of dependence and affects social structures by causing inequalities between the two parties in an exchange relation (Cropanzano and Mitchell, 2005; Najafi-Tavani et al., 2020). The higher the power imbalance, the higher inequalities in a social relation, and hence the higher ability and responsibility of the “superior” party. SCD, a dependence relationship resulting from a customer’s passive choice, implies that he or she perceives the service provider of power and capability and therefore holds them more responsible for the whole service process (Len et al., 2006; Najafi-Tavani et al., 2020). Thus, we hypothesize:

*H4*: Switching-cost dependence is positively related to internal attribution.

### The moderating role of relationship length

Relationship length refers to how long the customer had been dealing business with the service provider (Hui et al., 2011; Wang et al., 2021). Previous studies on relationship length show that *new* customers differ from those who have long been clients of their company (*old* customers). For instance, Coulter and Coulter (2002) find that the effect of offer-related service characteristics on trust increases as the relationship continues; however, the effect of “person-related” service characteristics diminishes over time. Similarly, Kumar et al. (2003) argue that relationship length does not always enhance the relationship or emotional attachments customers choose to have with their service providers. Chiao et al. (2008) distinguish relationship orientations and suggest that relationship length has a significant moderating role on the impact of satisfaction and trust on customer loyalty. Wang et al. (2021) find that the longer the relationship length is, the higher of optimal customer participation level with customer satisfaction and affective commitment.

Service relationship evolves with service encounters. At the early stages of a service relationship, when customers often have limited information or experience with the service offering and provider, prior relationships have pronounced effects in the attribution process to influence forgiveness. As the relationship ages, customers gain more information and become more knowledgeable about the service offering and the company, increasing the trust and reducing the perceived risk of their relationship with the service provider (Dagger and O’Brien, 2010; Wang et al., 2021). Accordingly, we propose:

*H5*: As the length of the relationship increases, the effect of RVD on external attribution decreases.

*H6*: As the length of the relationship increases, the effect of SCD on internal attribution decreases.

**Figure 1:**
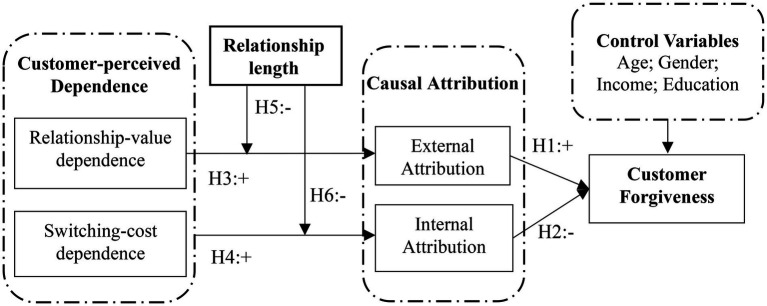
Conceptual model of our research.

Therefore, we propose the following conceptual model to reveal the underlying mechanism from customer-perceived dependence to forgiveness after service failure occurs, as illustrated in Figure 1.

## Methodology

Following previous studies on service failure and customer behaviors (Patterson et al., 2006; Barakat et al., 2015; Béal et al., 2019), we employ the mixed research design in the present study. Study 1 is an experiment with the scenario stimulus method, which is useful to investigate complex concepts (such as customer-perceived dependence) that are not easily operationalized in the real world (Eroglu, 1987). It has been extensively validated in previous research (e.g., McCollough et al., 2000; Smith and Bolton, 2002; Yim et al., 2008). We apply the quantitative survey method in study 2, which can help to identify the meanings and the relationships among all variables and develop a validated framework and theoretical contribution (Saunders et al., 2009). We discuss the study design in detail in the following sessions.

### Study 1: Experiment

The purpose of study 1 is to validate the existence of customer-perceived dependence in the B2C service context empirically and test the validity of the model hypothesis preliminarily. More specifically, we are looking forward to investigating four distinct types of customer-perceived dependence *via* a 2(relationship-value dependence: high vs. low) X 2(switching-cost dependence: high vs. low) experiment design.

#### Sample and data collection

One hundred and forty commercial bank customers in Mainland China were recruited to participate in an online experiment. To manipulate the different dependence statuses, participants were asked to read the assigned scenario that presumably describes their relationship with the bank and randomly assigned to one of four virtual stimulus groups for a 2(relationship-value dependence: high vs. low) X 2(switching-cost dependence: high vs. low) manipulation. In our experiment, we designed four different scenarios (see Appendix) to stimulate the corresponding dependence types, which are “high RVD and high SCD” dependence, “high RVD and low SCD” dependence, “low RVD and high SCD” dependence and “low RVD and low SCD” dependence. After that, participants were asked to complete a seven-item (three-items for RVD and four-items for SCD) measure adopted from Scheer et al. (2010) and Scheer et al. (2015) to indicate the dominant dependence component.

Next, participants were asked to read Part II scenario that describes a bank service failure and then reported their agreement with a 5-item statement pertaining to their forgiveness intention for the service failure. To examine the mediating role of causal attribution in the service failure context, participants were asked to complete an index of causal attribution borrowed from Folkes (1984) and Hess (2008), which contains six items of two dimensions: external attribution (EA-including three items) and internal attribution (IA-including three items). All items were measured on a seven-point Likert scale.

#### Results and discussion

##### Validity and reliability

After excluding cases with significant missing data or errors, our new sample consists of 138 valid participants, among whom are 79 men and 59 women. We checked the reliability of scales using Cronbach’s alpha and composite reliability (CR) and the validity of scales using CFA and AVE. Results indicate adequate reliability (α-RVD = 0.804; α-SCD = 0.836; α-EA = 0.646; α-IA = 0.623; α-*CF* = 0.934) and validity.

##### Manipulation check

The four scenarios in Part I were used to stimulate four dependence relationships, respectively. As expected, all manipulations have been successful (shown in Table 1). For group 1, RVD and SCD levels perceived by participants are not significantly different, and both are higher than the total average (*n* = 33; Mean-RVD = 6.55, Mean-SCD = 6.42). For group 4, participants indicated both RVD and SCD levels were lower than the total average (*n* = 35; Mean-RVD = 4.22, Mean-SCD = 3.94). Group 2 and 3 participants showed a dominant dependence relationship as desired.

**Table 1:** Manipulation check of dependence.

	RVD	SCD	T	Sig.	Mean-value (*n* = 138)		T	Sig.
Condition 1: High RVD and High SCD	6.55	6.42	1.14	0.262	RVD	5.34	17.40	0.000^***^
					SCD	4.96	19.81	0.000^***^
Condition 2: High RVD and Low SCD	5.79	3.96	6.37	0.000^***^	RVD	5.34	2.43	0.021^**^
					SCD	4.96	−3.37	0.002^***^
Condition 3: Low RVD and High SCD	4.89	5.61	−3.85	0.001^***^	RVD	5.34	−1.90	0.067^*^
					SCD	4.96	3.10	0.004^***^
Condition 4: Low RVD and Low SCD	4.22	3.94	1.002	0.323	RVD	5.34	−3.72	0.001^***^
					SCD	4.96	−3.42	0.002^***^

* *p* = 0.1;

** *p* = 0.05;

*** *p* = 0.01.

##### Experience results

Participants’ indication of attribution is illustrated in Table 2. When both RVD and SCD are high or low, participants may go through a complicated attribution process thus showing no clear tendency of EA or IA. The preliminary results supported that when customers perceived a dominant RVD relationship (“high RVD and low SCD” dependence), they tend to make external attribution, which positively leads to forgiveness. In contrast, customers who perceived a dominant SCD relationship (“low RVD and high SCD” dependence) tend to make internal attribution with the reduced intention of forgiveness. Then, the next study will further investigate the only two scenarios with one dominant dependence relationship in our conceptual model to enhance the robustness of the above results.

**Table 2:** Comparison within groups by paired samples test.

	EA	IA	T	Sig.	*CF*
Group 1: High RVD and High SCD	5.27	5.17	0.74	0.467	4.48
Group 2: High RVD and Low SCD	4.71	3.89	2.69	0.011^**^	4.94
Group 3: Low RVD and High SCD	4.10	4.80	−2.92	0.006^***^	3.88
Group 4: Low RVD and Low SCD	4.43	4.27	0.579	0.566	4.59

** *p* = 0.05;

*** *p* = 0.01.

### Study 2: Survey

Upon validating the existence of customer-perceived dependence and its distinct types. Study 2 aims to investigate the relationship in our conceptual model and formally test all the hypotheses.

#### Sample and data collection

Our study recruited 540 randomly selected participants through an online marketing agency. When participants started with the questionnaire, they were asked to recall the actual relationship with the most commonly used commercial bank. Then they were exposed to a hypothetical service failure scenario (see Appendix) in which they were asked to imagine themselves as the victims to complete all questions. After excluding cases with significant missing data or errors, our sample in the current study consists of 428 participants, bringing the recovery rate close to 79.3%. The sample profile is described in Table 3. There are 21 commercial banks mentioned during the survey, covering all major banks in Mainland China.

**Table 3:** Sample profile.

Participants demographics				Bank information					
		*N*	Perc. (%)	Names	*N*	Perc. (%)	Names	*N*	Perc. (%)
Gender	Male	230	53.74	ICBC	126	29.44	HXB	1	0.23
	Female	198	46.26						
	Total	428	100	CCB1	90	21.03	SPDB	4	0.93
Age	≤25 years old	150	35.05	BCL	50	11.68	HSB	1	0.23
	26–35 years old	223	52.10	ABC	58	13.55	CGFB	1	0.23
	≥36 years old	55	12.85	PSBC	15	3.50	PAB	1	0.23
	Total	428	100	CMB	29	6.78	CCB2	5	1.17
Income level	≤3,000 yuan	147	34.35	RCC	3	0.70	CAB	1	0.23
	3,001–5,000 yuan	251	58.64	BC	26	6.07	HFB	1	0.23
	5,001–8,000 yuan	26	6.07	CMBC	4	0.93	JSCB	1	0.23
	≥8,001 yuan	4	0.93	IB	6	1.40	ZYB	1	0.23
	Total	428	100	CEB	4	0.93	Total	428	100

#### Measurement

Our questionnaire was designed based on existing literature in service failure and relationship marketing fields. Depth interviews and pre-tests were conducted to assess the survey format and improve measures before formal data collection. Every item was reciprocally translated between English and Chinese to ensure content validity. For customer forgiveness, we constructed our operationalization for emotional forgiveness and decisional forgiveness using a five-item scale borrowed and edited from the work of McCullough et al. (1998) and Tsarenko and Tojib (2012). Customer-perceived dependence measurement was developed based on the work of Scheer et al. (2010 and 2015), including three items for RVD and four items for SCD. The scale of attribution is adapted from the study of Folkes (1984) and Hess (2008), which is a six-item measurement with two dimensions: external attribution (three items) and internal attribution (three items). Control variables include gender, age, education, and income. All items were measured on a seven-point Likert scale, and the measurements of all constructs are described in Table 4.

**Table 4:** Construct reliability and validity analysis.

Construct/items	Loading
**Relationship-value dependence** **Cronbach α = 0.775, composite reliability = 0.78, AVE = 0.54**	
Compared to other banks, you can get more VIP privileges with the gold card provided by the bank you indicated above	0.744
The VIP privileges associated with the gold card mentioned above are difficult to obtain from other banks	0.705
Choosing the gold card mentioned above is very important for you to get the desired VIP privileges	0.721
**Switching-cost dependence** **Cronbach α = 0.873, composite reliability = 0.88, AVE = 0.65**	
If you cancel the gold card mentioned above, you will lose the points, special discounts and other card benefits	0.812
If you cancel the gold card mentioned above, you will have to take time and efforts to seek for and evaluate an alternative card	0.845
If you cancel the gold card service mentioned above, you will have to incur monetary cost to find an alternative card	0.752
If you cancel the gold card service mentioned above, you will have to spend time and effort to learn to adapt to the alternative card	0.808
**External attribution** **Cronbach α = 0.609, composite reliability = 0.78, AVE = 0.55**	
This service failure is caused by an external network attack	0.773
This service failure is accidental and non-enduring	0.853
This service failure is uncontrollable by the bank	0.568
**Internal attribution** **Cronbach α = 0.698, composite reliability = 0.82, AVE = 0.60**	
This service failure is caused by the internal website issues	0.773
This service failure is persistent and may occur again in the future	0.828
This service failure can be predicted and prevented by the bank in advance	0.718
**Customer forgiveness** **Cronbach α = 0.853, composite reliability = 0.90, AVE = 0.63**	
You will stop purchasing financial products from the bank mentioned above	0.764
You will not trust the financial products anymore from the bank mentioned above	0.872
You will cut off the ties with the bank mentioned above	0.785
You want to get compensated for the service failure by the bank mentioned above	0.775
You want the bank mentioned above to be penalized for the service failure	0.778
**Relationship length**	
How long have you had the service provided by the bank you mentioned above?	/
Control variables	/
Please select your gender;	/
Please fill out your age;	/
Please select your education degree;	/
Please select your approximate income level	/
χ^2^/df = 1.594, GFI = 0.955, AGFI = 0.931, IFI = 0.980, TLI = 0.972, CFI = 0.980, RMSEA = 0.037	

#### Results

##### Validity and reliability

Before testing our hypotheses, we access the reliability and validity results.

First, the reliability of scales is measured by Cronbach’s alpha and composite reliability (CR). As summarized in Table 4, all values of Cronbach’s alpha range from 0.609 to 0.873 and all values of CR range from 0.78 to 0.90, which indicates adequate reliability of our measurement scales (De Matos et al., 2013; Sengupta et al., 2015).

Second, we take advantage of the maximum likelihood approach in CFA to evaluate the convergent validity of each measurement scale. As shown in Table 5, all indicators in their respective constructs have statistically significant (*p* < 0.05) factor loadings from 0.568 to 0.872, which supports the convergent validity of all theoretical constructs (Sengupta et al., 2015). Next, the convergent validity is also accepted because the average variance extracted (AVE) of each construct exceeds the recommended minimum value of 0.5 (De Matos et al., 2013).

**Table 5:** Mean, standard deviations, and correlations of the constructs.

Variables	Mean	S.D.	RVD	SCD	EA	IA	*CF*	RL
RVD	5.00	1.22	**0.73**					
SCD	4.89	1.40	0.662^**^	**0.81**				
EA	4.01	1.20	0.230^**^	0.211^**^	**0.74**			
IA	4.61	1.19	0.092	0.157^**^	−0.083	**0.77**		
*CF*	4.27	1.34	0.201^**^	0.159^**^	0.267^**^	−0.413^**^	**0.79**	
RL	3.07	0.92	0.057	0.087	0.013	0.160^**^	−0.061	1

RVD, relationship value dependence; SCD, switching cost dependence; EA, external attributions; IA, internal attribution; CF, customer forgiveness; RL, relationship length. Bold value indicates square root of AVE is on the diagonal.

** *p* < 0.005;

*** *p* < 0.001.

Third, we compare the average variance extracted (AVE) of each pair of constructs with their squared correlation and the results in Table 5 suggest satisfactory discriminant validity. In addition, we conduct Harman’s single-factor test as a *post hoc* statistical analysis to examine any potential threat of common method bias (CMV). It shows that five factors emerged from the analysis while no single general factor accounts for most of the variance in those variables. Five distinct factors have eigenvalues greater than 1.0 and account for 65.82% of the total variance. The first factor explained 25.85% of the variance, which indicate that CMV should not be a significant concern in our data set.

##### Test of model and hypotheses

We use the structural equation model to test our theoretical framework and hypotheses. Overall, our theoretical model has a very good fit with the data (χ2/df = 1.594, GFI = 0.955, AGFI = 0.931, IFI = 0.980, TLI = 0.972, CFI = 0.980, RMSEA = 0.037).

**Figure 2:**
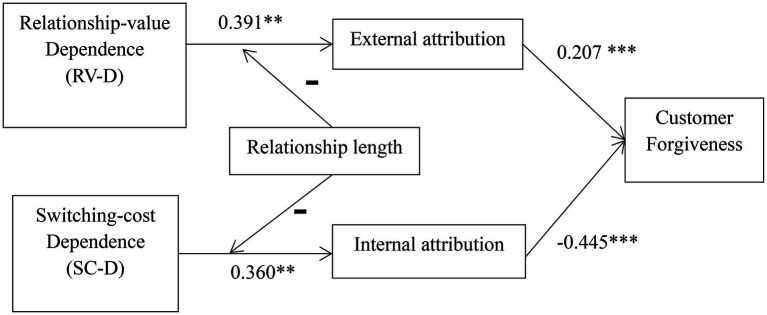
Results of path estimates analysis (^**^p < 0.005, ^***^p < 0.001).

All our path estimates are presented in Figure 2. It shows that the RVD has a positive influence on external attribution (β = 0.391, *p* < 0.05), whereas SCD has a positive impact on internal attribution (β = 0.360, *p* < 0.05), supporting H3 and H4. At the same time, the external attribution has a positive effect on customer forgiveness (β = 0.207, *p* < 0.01) while the internal attribution has a negative effect on customer forgiveness (β = −0.0445, *p* < 0.01), supporting H1 and H2.

Findings indicate that RVD and SCD not only have different total effects on customer forgiveness but also go through differential causal attribution routes. Specifically, RVD is directly linked with external attribution, which further positively impacts customer forgiveness. Similarly, SCD directly affects internal attribution, which further leads to a negative impact on customer forgiveness. There is no direct effect of RVD or SCD on customer forgiveness. (Direct and indirect effect results are presented in Table 6).

**Table 6:** Direct and indirect effects.

Paths	Direct effect	Indirect effect	Total effect
RVD → external attribution	0.391	**-**	0.391
External attribution → customer forgiveness	0.207	**-**	0.207
RVD → customer forgiveness	**-**	0.08	**0.08**
SCD → internal attribution	0.36	**-**	0.36
Internal attribution → customer forgiveness	−0.445	**-**	−0.445
SCD → customer forgiveness	**-**	−0.16	**−0.16**

Bold value indicates direct and indirect effect results are presented.

We examine the moderating effects of relationship length on every path using multi-group analysis. Following Germain et al.’s (2008) practice, we use a procedure to divide the sample into two based on the composite score of relationship length. Then we conducted multi-group and structural path analyses to investigate the moderating effect of a short-term and long-term relationship. Results summarized in Table 7 show that the effects of RVD on external attribution and SCD on internal attribution are significantly different across the short-term and long-term groups, which supported H5 and H6.

**Table 7:** Summary of multi-group analysis.

		Parameter estimates		Tests for invariance		Hypotheses
		Short-term relationship (<5 years)	Long-term relationship (>5 years)	Chi-square difference	Significance	
**Paths**	**RVD → EA**	**0.352** ^***^	**0.093**	CMIN = 305.5 DF = 226	0.000	**H5 supported**
	**SCD →IA**	**0.175** ^***^	**0.059**			**H6 supported**
	EA → *CF*	0.512^***^	0.080			
	IA → *CF*	−0.449^***^	−0.389^***^			

*** *p* < 0.001.

## Conclusion and discussion

Understanding customer forgiveness will benefit organizations in actions designed to restore a positive relationship with customers, beyond the notion of customer retention (Tsarenko and Tojib, 2011; Yang and Hu, 2021). In the current study, we investigate the underlying relationship between customer-perceived dependence, causal attribution, and customer forgiveness, as well as the moderating effect of relationship longevity. Our results show an indirect effect of the dependence relationship on customer forgiveness through differential attribution paths. Specifically, relationship-value dependence (RVD), rooted in ongoing net benefits, is more likely to trigger external attribution that positively leads to forgiveness. In contrast, switching-cost dependence (SCD) rooted in latent cost is related to internal attribution that negatively impacts forgiveness. Furthermore, the relationship between dependence and causal attribution is negatively moderated by relationship length measured in the duration of how long the customer had been dealing business with the service provider.

### Theoretical contributions

Our study advances the understanding of consumer forgiveness by elucidating the underlying mechanism of the dependence relationship in the service failure context. Our study answers the calls of Cambra-Fierro et al. (2015) to study the conceptualization of relationships further to advance the understanding of its impact on forgiveness. As dependence becomes more prominent in the B2C service market, it’s worth investigating whether/how customers’ dependence on a service provider would lead to their forgiveness as a positive coping strategy after service failure encounters. By providing empirical grounds in such accounts, we set from this new theoretical angle meanwhile elaborate the boundary conditions that govern customer forgiveness.

The current study also promotes the theoretical understanding of dependence by shedding light on two different sides of a customer’s motivational investment. The findings would enrich the extant dependence literature by providing empirical evidence beyond dependence extent, symmetry, or directivity. Scheer et al. (2015) noted that the inherent ambiguity in general dependence measures has failed to capture a comprehensive understanding of all aspects and sources of dependence. As such, our framework investigates a customer’s RVD and SCD orientation simultaneously, and the findings suggest RVD is related to external attribution, whereas SCD is associated with internal attribution.

Last, we extend the service literature by bridging the research gap between relationship framed post-transgression reactions and causal attributions. When customers experience unsatisfactory services, they seek to understand why because they sense a need to understand, control, and predict their environment (Weiner, 2000; Ho et al., 2020). And how they attribute the fault after a service failure, especially when the root cause is not evident, will largely determine their post-transgression reactions. In service recovery research, one stream focuses on post-transgression negative reactions to service failure outcomes, while the other on else determinant factors such as causal attributions and customer’s prior relationships. There deserves more attention in a holistic study of forgiveness, causal attributions, and customer–firm prior relationships (Sinha and Lu, 2016). To this end, our study attempted to elicit the role of the individual attribution process in mediating the effect of the dependence relationship on customer forgiveness. Our findings also contribute to the attribution literature by exploring the dependence relationship as a new antecedent of the attribution process.

### Managerial implications

The industry-wide digitalization and Covid-19 pandemic bring firms opportunities and challenges. While leveraging technologies to better engage with customers and boost firm performance, companies also face more significant risks of service failures associated with the technology. Our conceptualization of customer-perceived dependence depicts customers’ reaction mechanisms in the event of service failure. Practitioners can develop marketing strategies accordingly by building upon the knowledge of why customers choose to remain in the service business relationship. Moreover, our study has shown that RVD is positively related to forgiveness because customers tend to make external attribution, and SCD is the contrary. Managers can leverage new technology to better understand and segment customers based on their dependence type, taking proactive approaches accordingly in response to a service failure. Last but not the least, as the linkage between dependence and causal attribution is negatively moderated by patronage length, managers can implement service recovery strategies for short-term and long-term relationships with customers.

### Limitations and future research

Our study has a few limitations. Firstly, to remain a parsimonious model, we constructed customer-perceived dependence from a broader relational benefits and costs view, without considering social, psychological, or other customizable factors that may also form dependence. Thus, future research can further explore the conceptualization and measurement realization of the customer-perceived dependence construct. Secondly, some literature argues that forgiveness is a continuous process in which consumers’ cognitive, affective, and behavioral aspects all play crucial roles. The interactive impact of attribution and any other factors should be examined in future studies, such as relational norms, failure severity, perceived fairness, recovery strategies, and customer emotions. Finally, our research finding is limited in scope as it was conducted in the bank industry of the Chinese market. Future research should look into other industry contexts and different cultures or countries.

## Data availability statement

The original contributions presented in the study are included in the article/supplementary material, further inquiries can be directed to the corresponding author.

## Author contributions

XC and SG were responsible for the designing and writing. XC and JX were responsible for the data analysis. SG and SH were responsible for English language editing. All authors contributed to the article and approved the submitted version.

## Funding

This research was supported by the Humanities and Social Science Grant from the Ministry of Education, China (grant #21XJC630001) and the National Natural Science Foundation of China (grant #71772142).

## Conflict of interest

The authors declare that the research was conducted in the absence of any commercial or financial relationships that could be construed as a potential conflict of interest.

## Publisher’s note

All claims expressed in this article are solely those of the authors and do not necessarily represent those of their affiliated organizations, or those of the publisher, the editors and the reviewers. Any product that may be evaluated in this article, or claim that may be made by its manufacturer, is not guaranteed or endorsed by the publisher.

## Appendix

### Part I. Virtual stimulus scenarios: Four different types of customer-perceived dependence in study 1

**Table:**

**Condition 1: High RVD and High SCD**
You are a VIP gold-card holder of a national commercial bank, which is named Xinyuan bank. As a VIP member, you have always enjoyed the card member benefits such as express line checkout, airport VIP lounge, holiday gifts and so on. With Xinyuan bank, you have also been assigned a personal wealth management financial advisor, who understands your financial status and goals, and has guided you on planning customized financial strategies to achieve your long-term and short-term investment objectives. On the other hand, you have had spent a great deal of time and efforts in learning Xinyuan’s financial products, service policies, and even established a good relationship with the financial advisor. Being a VIP customer, you have always maintained high value assets and a high volume of transactions. In your opinion, the VIP gold-card membership status of Xinyuan bank is important for your work and life. Once you lose it, you will have to incur additional time and effort, and even monetary costs in order to get the same service at other banks. For such reasons, you would like to remain the VIP gold-card holder membership.
**Condition 2: High RVD and Low SCD**
You are a VIP gold-card holder of a national commercial bank, which is named Xinyuan bank. As a VIP member, you have always enjoyed the card member benefits such as express line checkout, airport VIP lounge, holiday gifts and so on. With Xinyuan bank, you have also been assigned a personal wealth management financial advisor, who understands your financial status and goals, and has guided you on planning customized financial strategies to achieve your long-term and short-term investment objectives. Being a VIP customer, you have been acquainted with Xinyuan’s financial products and service policies, which are similar to that of other banks. In your opinion, Xinyuan’s VIP experience is better than other competitors,’ and is important for your work and life. In order to continue enjoying the card member benefits, you would like to remain the VIP gold-card holder membership.
**Condition 3: Low RVD and High SCD**
You are a VIP gold-card holder of a national commercial bank, which is named Xinyuan bank. Similar to other banks, the VIP cardholder status of Xinyuan bank is not easy to obtain, which requires a certain value of assets and volume of transactions. Being a VIP customer, you have had spent a great deal of time and efforts in learning Xinyuan’s financial products, service policies, and even established good relationships with the financial advisor. However, the VIP status at Xinyuan bank did not offer you benefits that suit your needs or service significantly better than other banks. It’s still hard to give up the VIP status since you may have to incur additional time and effort, and even monetary costs in order to get service at other banks.
**Condition 4: Low RVD and Low SCD**
You are a VIP gold-card holder of a national commercial bank, which is named Xinyuan bank. As a VIP member, you have been acquainted with Xinyuan’s financial products and service policies, which are similar to that of other banks. Being a VIP customer, however, you did not receive benefits that suit your needs or service better than that of other banks. In your opinion, even if the VIP status is lost, you can also easily get the same services from other banks.

### Part II. Service failure scenarios in both study

Recently, you plan to buy a new financial investment plan. You contact your personal financial consultant, hoping to be recommended for some suitable financial products. Based on your budget, risk preferences and expected return, you are recommended for a variety of financial products. After careful comparison, you picked one product and received all purchase information in detail. However, when you tried to buy it online before the deadline, you were unable to refresh the page to complete the purchase. As the purchase deadline approaches, you called the bank customer service to inquire, but the reply is “part of our website functions have not been restored due to the recent cyber hacking attacks. We are trying to fix the problem as we speak, please accept our sincere apologies.” Frustrated, you end up missing the opportunity to buy the financial products.
